# The origins and growth of the Meatless Monday movement

**DOI:** 10.3389/fnut.2024.1283239

**Published:** 2024-03-14

**Authors:** Richard D. Semba, Peggy Neu, Pamela Berg, Jamie Harding, Shawn McKenzie, Rebecca Ramsing

**Affiliations:** ^1^Johns Hopkins Center for a Livable Future, Bloomberg School of Public Health, Baltimore, MD, United States; ^2^The Monday Campaigns, New York, NY, United States

**Keywords:** climate change, diet, greenhouse gas production, meat, protein, vegetarianism

## Abstract

Meatless Monday is a global movement that encourages people to reduce meat in their diets for their own health and the health of the planet. We conducted a comprehensive review of primary and secondary sources and archival material documenting the origins, historical roots, and growth of Meatless Monday and simultaneous developments in public health. Sources for the paper included publications of the US Food Administration and articles and media identified using searches of ProQuest Historical Newspapers, Newspapers.com Academic, ProQuest US Newsstream, ProQuest Canadian Newstream, ProQuest International Newsstream databases, and Google.com. Meatless Monday was conceived by the advertising executive and public health advocate Sid Lerner in 2003, inspired by the meatless days observed during World War I and II. Meatless Monday grew steadily from 2003 to 2023 through advocacy by food writers, talk show hosts, and celebrity chefs, and through participation by schools, cities, restaurants, corporations, and institutions worldwide. School systems began to observe Meatless Monday, such as Baltimore City Public Schools in 2009 and New York City Public Schools in 2019. Meat-Free Monday campaign was launched by Paul McCartney and his daughters in 2009 in the United Kingdom. The Humane Society of the United States became an advocate for Meatless Monday and helped institute it in >200 US school systems. From 2003 to 2023, Meatless Monday spread to over 40 countries and was observed in public schools in countries such as Brazil, Ireland, and Belgium. Findings regarding high meat consumption and its adverse effects on health, high greenhouse gas production and environment degradation, and problems with animal welfare under conditions of industrial food animal production emerged during the same period and influenced many to advocate Meatless Monday. Meatless days of World War I and II were driven by patriotic motivations to provide food for the US troops and the Allies in Europe, whereas motivations for observing Meatless Monday were largely related to concerns regarding personal health, the environment, and animal welfare. Meatless Monday grew from relatively humble origins to a highly recognized worldwide movement with wide appeal as a way to begin reducing meat consumption for personal and planetary health.

## 1 Introduction

Food systems contribute to an estimated 30% of global greenhouse gas emissions (GHGe) (1). Livestock, which include primarily ruminant (cattle, lamb) and monogastric (pigs, chicken) animals, account for 14.5% of total human-induced GHGe (2) and 30% of global anthropogenic methane emissions (3). Beef is by far the food with the greatest climate footprint (4, 5). A potential strategy to reduce GHGe and alleviate global warming is to reduce meat consumption and shift to primarily plant-based diets (6–10). A shift from meat to plant-based dietary patterns also reduces the risk of adverse chronic disease outcomes such as type 2 diabetes (11, 12), cardiovascular disease (12, 13), frailty (14), and mortality (12, 13, 15). Whether dietary recommendations or public health campaigns can convince people to decrease meat consumption remains unclear (16). Among the most visible public health strategies of reducing meat consumption has been Meatless Monday, a global movement that encourages people to reduce meat in their diets for their own health and for the health of the planet (17).

The Meatless Monday campaign was founded by the advertising creative director and public health advocate Sid Lerner (17). The Center for a Livable Future of the Johns Hopkins Bloomberg School of Public Health provided scientific and technical expertise to the campaign. The relationship between the Meatless Monday campaign and the Center for a Livable Future has been strong and continuous since its founding. Meatless Monday had its historical origins in meatless days that were observed in the US partly as a goodwill effort to provide meat for Allies in Europe during World War I and II. Over the last two decades, the reach and contributions of Meatless Monday have spread across the United States and worldwide. The definition of “meat” has varied over time. During World War I and II, “meat” meant beef, lamb, and pork (18). During the first years of the Meatless Monday campaign, “meat” included all livestock meat, i.e., chicken, pork, lamb, and beef (17). Around 2010, fish was also included in the term “meat” in materials distributed by the Meatless Monday campaign (Pamela Berg, personal communication).

This paper aims to address a gap in the literature regarding the history of the Meatless Monday campaign. The goal of this paper is to present the history of Meatless Monday since its founding in 2003, the history of meatless days in World War I and World War II, and the expansion of the Meatless Monday movement from 2003 onwards. The Meatless Monday campaign grew during parallel developments in public health, such as increased concerns about the link between red and processed meat with cardiovascular disease and cancer, new findings on climate change that identified livestock as a major contributor to greenhouse gas production, and alarm raised about animal welfare in “factory farms.” The emerging findings on health, climate change, and animal welfare prompted many individuals and organizations to become advocates for Meatless Monday. We conclude with a discussion about the impact of the Meatless Monday campaign and gaps in research.

## 2 Methods

The historical sources for the paper included official publications of the US Food Administration and newspaper articles, online publications, newswires, and blogs identified using searches of ProQuest Historical Newspapers (19), Newspapers.com Academic (20), ProQuest US Newsstream (21), ProQuest Canadian Newstream (22), ProQuest International Newsstream databases (23) and Google.com using the terms “meatless monday,” “meat-free monday,” and “meatless day.” The archive of the Meatless Monday campaign was also used as a source for blogs and newswires (24). The search was limited to the time range of January 1, 2002 to December 1, 2023. Most of the available newspaper and periodical databases are based upon publications from the English-speaking world, but articles in Spanish, Portuguese, and French were also searched. Meatless Monday is compared and contrasted with its antecedents of meatless days during World War I and II. The growth of the Meatless Monday movement is presented in the context of parallel developments in medicine, public health, environmental science, and animal welfare that were occurring during the same period. Examples are given where these parallel developments influenced attitudes about the implementation of Meatless Monday. A figure showing Meatless Monday activities from 2003 to 2023 was developed using ArcGIS Pro software (Environmental Systems Research Institute [ESRI], Inc., Redlands, California). Meatless Monday sites were geocoded with the ArcGIS World Geocoding Service (ESRI) and then displayed by the year the Meatless Monday program began. The country boundaries on the figure were obtained from the United Nations. The figure uses the Robinson projection. Meatless Monday programs and locations were provided by the Meatless Monday Campaign based upon news releases, blogs, newspaper articles, personal communications, blogs, and web searches. A table that summarizes the approaches to meatless days was created to make a historical comparison (25) between the period of World War I and II with the Meatless Monday campaign (2003–2023). The table is a subjective historical interpretation by the authors of the referenced works. It is meant to highlight similarities and differences between these two historical periods. Salient points of comparison were selected by the lead author (RDS) with two rounds of revisions consisting of additions by the coauthors. There were no discrepancies in agreement during the revisions, and adjudication was not required.

## 3 Sid Lerner and the concept of the Meatless Monday campaign

Meatless Monday was conceived by Sid Lerner in 2002 during a meeting with Alfred Sommer, Dean of the Johns Hopkins Bloomberg School of Public Health, and Robert Lawrence, the Director of the Center for Livable Future, Associate Dean, and Professor at the Johns Hopkins Bloomberg School of Public Health in Baltimore (Robert Lawrence, personal communication). At the time, Lerner thought that an important avenue in addressing the problems associated with industrial food animal production was to identify ways to help people reduce their meat consumption. He was especially concerned about an excess of saturated fat in the diet. He was also alarmed about his own health, as his doctor had started him on a statin to lower his cholesterol. “Everybody was talking about fat and cholesterol,” said Lerner, “and meat and fat in the diet.” “I asked Bob [Robert Lawrence], ‘what is too much?’ (26) Lawrence replied that the Surgeon General, US Department of Agriculture (USDA), and others advised that Americans “…were eating over 15% of what we ought to be eating of this fat heavy diet which led to heart disease, cancer, stroke and diabetes. So how do you cut down 15%?” asked Lerner. It occurred to him that 15% of 21 meals in a week is three meals, or one day’s worth. “So make it simple,” said Lerner, “just one day a week knock off the fat and meat in the diet. You sort of make a dent in it, as you should, but an easy way. Just one day a week to catch up on other good things that aren’t meat in the middle of the plate. So that was the beginning of Meatless Monday” (27).

Lerner was an influential figure during the “Mad Men” era of advertising on Madison Avenue, overseeing advertising campaigns for well-known brands such as Texaco and Maxwell House and motivating Americans to purchase common household products such as toilet paper with the memorable “Please Do not Squeeze the Charmin” featuring Mr. Whipple (28). Lerner found inspiration for Meatless Monday from his childhood recollections of “Meatless Tuesdays” during World War II and its earlier roots in World War I (26). The practice of meatless days was widespread and familiar to nearly all families growing up during the world wars in North America and Europe (18). When the Meatless Monday campaign was launched in 2003, it was endowed with a rich historical record that demonstrated meatless days were feasible in the past.

## 4 Meatless days during World War I

This section refers largely to how meatless days began in the US during World War I. After World War I broke out in Europe in August 1914, the US remained neutral under President Woodrow Wilson. Food production in Europe became greatly compromised due to several factors. There was a shortage of farm labor, since sixty million men of the nations in.

conflict were diverted from farms to the military (18). There was diminishing availability of fertilizer and feed (18). Much of the farm and cattle-grazing land was devastated by fighting (18). Serviceable farm machinery became scarce (18). Food supplies for the Allies from Russia, Rumania, Bulgaria, Serbia, and Turkey had been cut off by the Central Powers (Germany, Austria-Hungary, Bulgaria, and the Ottoman Empire) (18). There was a shortage of animal feed due to poor harvests, loss of transport ships due to sinkings from German torpedoes, and diversion of ships to support the war efforts. Among both the Allies and Central Powers, meatless days were enforced to conserve food for their respective armies. Austria and Italy had two meatless days per week. Bulgaria had three meatless days per week (29). One meatless day per week was observed in England (30). Two meatless days per week were observed in France in 1917 and extended to three meatless days per week in 1918 (31).

After German submarines sank U.S. merchant ships, President Woodrow Wilson called for a declaration of war against Germany in a joint session of Congress on April 2, 1917. Upon entry into the war, President Wilson declared that the first task was to “supply abundant food” to the Allies (32). The food supply to the American people was generally produced in a surplus, except for sugar, vegetable oils, and coffee, of which imports were decreased during the war (33). In May 1917, President Wilson outlined a food control program, deemed necessary to provide an adequate distribution of food for the US civilian population, US military, and the Allies and to prevent exorbitant prices of food in the US, notably meat, sugar, and wheat, due to hoarding, speculation, and erosion of the balance between supply and demand. He appointed Herbert Hoover as Food Administrator, an apt choice since Hoover had previously organized the Committee for Relief in Belgium – which fed nearly nine million people in Belgium and German-occupied northern France – and had garnered strong experience with the logistics of food relief (34). As Food Administrator, Hoover was responsible for directing the US Food Administration. On August 10, 1917, Wilson signed the Food Control Bill, a law that created the Food Administration. J. Ogden Armour, the owner of Armour Company, the largest meatpacker in the US, advocated government control of all provisions so that the people of the US and the Allies would have enough food (35). Prior to any actions by the Food Administration, meatless days were promoted in some localities, such as Parkersburg, West Virginia (36), Chicago (37), and Jackson, Mississippi (38), and railroad companies in the US (39). Canada was also supplying meat to the Allies and declared two meatless days in May 1917 (40).

Since the Food Administration lacked the authority to ration foods, its efforts to conserve food and prevent food waste depended primarily upon the patriotic cooperation of the people to ensure its success (18). The slogan “Food Will Win the War” was announced by Hoover for the campaign to enlist every “housewife” in the US to participate in two meatless days by signing a conservation pledge card (41). October 9, 1917, was declared the first beefless Tuesday nationwide by the Food Administration, a measure that was widely observed across the country (42). The Hotel and Restaurant Conservation Committee of Food Administration agreed that restaurants and hotels would observe two meatless days weekly – Tuesdays and Fridays (43). In New York City alone, according to the Food Administration observation of meatless Tuesday saved 116 tons of meat in 1 week, with estimates that 300 tons of meat would be saved with scaled up participation (44). By November 1917, the Food Administration reported that one out of every three families in the US pledged support for their plan of voluntary food conservation (45). The food situation in Europe was becoming increasingly dire, and in early 1918, the Food Administration asked households across the US to pledge to observe a meatless Tuesday, a porkless Tuesday and Saturday, as well as a meatless meal each day (46). A humorous poem that poked fun at the Food Administration became well known across the US: “My Tuesdays are meatless, My Wednesdays are wheatless; I am getting more eatless each day. My home, it is heatless; My bed, it is sheetless…” (18).

**Figure 1:**
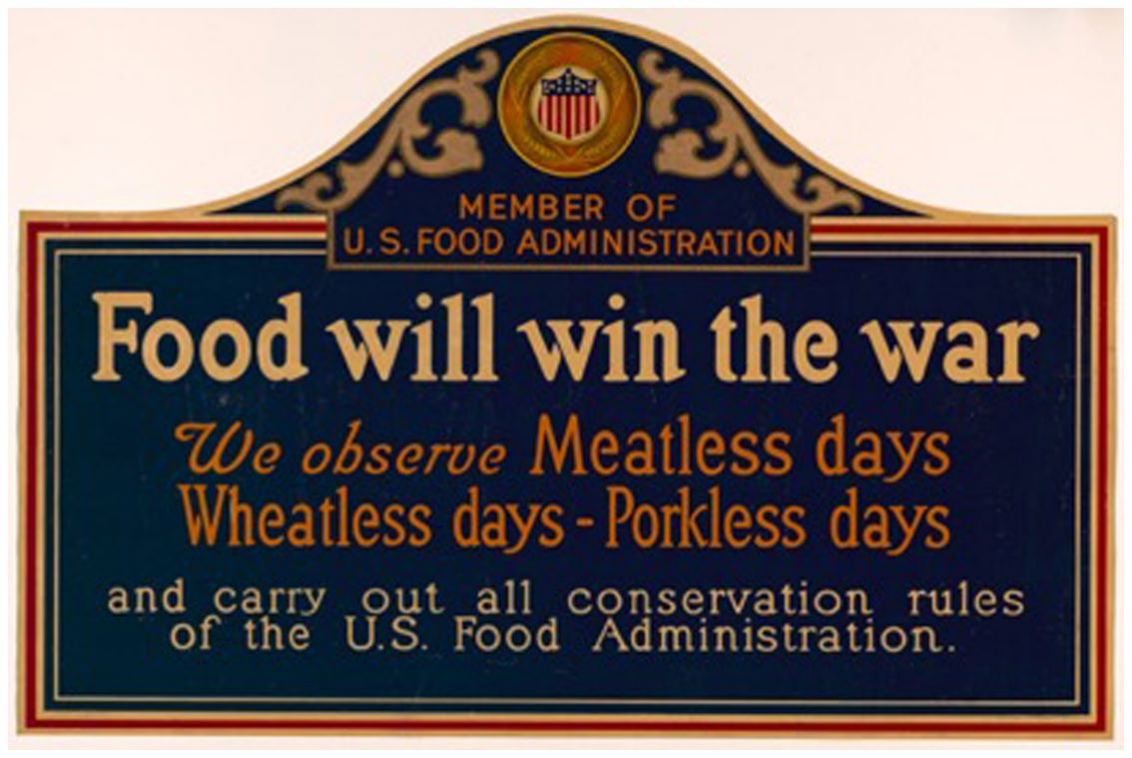
US Food Administration “Food will win the war” poster, for display in windows of partipating restaurants. New York American Lithograph Company, 1917. Library of Congress Prints and Photographs Division, Washington, DC. “Food will win the war” by New York: American Lithographic on Library of Congress, licensed under Public Domain.

The Food Administration advocated meatless days and the conservation of food through publication, posters (Figure 1), and advertising in newspapers, magazines, farm journals, trade journals, religious press, and libraries, a speakers’ bureau, and distribution of placards, slides, and motion pictures to movie theaters (18). Movie stars, such as Douglas Fairbanks and Mary Pickford, appealed to the public to support meatless days (18). Textbooks, such as *Food Saving and Sharing* for young children (47), *Food Guide for War Service at Home* (48) aimed at high school students, and *Food and the War: A Textbook for College Classes* (49) were widely distributed across the country (18). The nutritionist Mary Swartz Rose gave advice to “patriotic housewives” in *Everyday Foods in War Time*, which included an appendix with mostly meatless recipes (50). Rose advised that meat was only one of many protein-rich foods and was “no better than milk or eggs” and could easily be replaced by peanut butter, navy beans, or split peas (50). Furthermore, she noted: “Meat is produced at the expense of grain, which we might eat ourselves. And the production of meat is a very wasteful process” (50). A cookbook, *Foods That Will Win the War* provided meatless recipes for the home kitchen (51). War gardening efforts that promoted home gardening across the country raised awareness about supporting the Allies and led to greater consumption of vegetables (52). By the spring of 1918, the meat supply was increasing, allowing the US to supply the Allies with meat and ease some restrictions at home (53). The Food Administration announced that meatless Tuesdays would continue but the meatless meal and porkless Saturday would no longer be required since hogs became more available (53). Meatless days were temporarily suspended for 30 days on March 29, 1918 and never reinstated (33). Instead, consumers were advised to voluntarily limit their purchases and consumption of meat. No specific limits of purchase and consumption were made in this general recommendation. World War I ended on Armistice Day, November 11, 1918, but food conservation was still advocated to alleviate the serious food shortage in Europe (54).

The Food Administration facilitated a nearly eight-fold increase of pork and nearly ten-fold increase of beef exported from the US to Europe during the war and early post-war period. In the 2 years, 1917–1919, 2,340,705 tons of pork and 902,116 tons of beef were exported to Europe compared with 298,115 tons of pork and 93,187 tons of beef exported to Europe in the 2 years, 1912–1913, before World War I (18).

## 5 Meatless days during World War II

Meatless days returned to Europe with the outbreak of World War II. Hitler invaded Poland in September 1939, after which France and Great Britain declared war on Germany. In order to save meat for the army, France imposed meatless days on Monday and Tuesday at the beginning of the war (55) and added a meatless day on Friday in December 1939 (56, 57). By the spring of 1940, Belgium was observing meatless days on Monday (58), and Italy instituted three meatless days per week (59). The typical English dinner of roast beef became a rarity (60). Early in 1941, Lord Woolton, Minister of Food for Great Britain, asked the people of the US to reduce their consumption of certain foods such as meat, so that the surpluses could be sent to Great Britain (61). The US announced in October 1941 that it would help feed one-quarter of the population of Great Britain with food shipments, including 1.5 billion pounds of pork and lard in 1942, without causing shortages at home (62) as the US meat supply was at an all-time high (63). The USDA predicted there was no need to return to the “meatless, wheatless, or otherless days” of World War I (64).

President Franklin Delano Roosevelt raised the idea in 1942 that if a meatless day were observed 1 day per week in the US, it would free up 30 to 40 ships that could be used for military purposes instead of transporting meat from Argentina, New Zealand, and Australia to the US (65). The International Stewards and Caterers Association responded by adopting a resolution for Meatless Tuesdays. The association had 2,100 members in twenty-one cities, including New York, Philadelphia, Boston, Pittsburgh, Detroit, Cleveland, Cincinnati, St. Louis, Denver, Chicago, San Francisco, and Los Angeles (66). One meatless day per week was adopted by federal cafeterias in Washington, DC, in September 1942 (67) and by the Los Angeles public schools (68). The mayor of New York City, Fiorello La Guardia, asked the hotels and restaurants to make Tuesday a meatless day (69). Los Angeles adopted meatless Tuesdays at restaurants, schools, hotels, and clubs, with reported near 100% compliance in 1942 (70).

The US issued a War Food Communique warning that “meat rationing to begin on or about February 1, 1943” (71). The flyer, distributed door-to-door across the country, declared “Food is a Weapon of War!” Appealing to patriotism, the communique urged people who were accustomed to eating more than 2 ½ pounds of meat per week to cut back their consumption so that more meat could go to the Allies and troops (71). A complicated system of meat rationing was instituted by the US government in March 1943 that involved a point rationing system of meat to restaurants and booklets of ration stamps to individuals for meat, i.e., beef, lamb, and pork (72). New York City continued to observe meatless Tuesday in 1943, along with Philadelphia, Los Angeles, Boston, and San Francisco, but the rest of the US largely did not observe meatless days (73). When a new record in US meat production was reached in 1944, the annual consumption of meat averaged 154 pounds per person, the highest level of consumption since 1909 (74). As meat supplies were rising, meatless Tuesdays ended in New York and other cities in September 1945 (74). World War II ended on September 2, 1945, but the US public was asked to keep conserving food for post-war famine relief in Europe. In 1947, a US Gallup poll reported that 22% of respondents observed meatless Tuesdays as urged by the government, while 38% of respondents indicated that they were planning to follow it (75).

The experiences from World War I and II showed that meatless days could be widely observed by a large proportion of the population in the US and other countries at the time using an appeal to patriotism and a greater good. A common theme in the appeal for meatless days was that ordinary people in the US could help the Allies in Europe, who were poor and suffering much more than the privileged Americans (18, 47–49, 54, 62). Later in 2003, when the Meatless Monday campaign began, many of the generation of older people who lived through World War II era still remembered the meatless days they experienced in their youth (18, 26).

## 6 Initial developments in the Meatless Monday movement

Sid Lerner established Meatless Monday as a nonprofit public health initiative with the mission of helping people reduce meat and saturated fat in their diet (26). He hired a small team of experienced advertising professionals to promote Meatless Monday to consumers as well as organizations who could utilize the concept to accomplish their own goals, as he strongly believed in marketing best practices in public health (26). “There’s no ‘McDonald’s Day’ or ‘Coca-Cola Day’ because those guys are in your face all the time,” said Lerner. “We have to put a new face, mentality and drive behind public health communications and promotions.” (76). Lerner preferred “Meatless Monday” instead of “Meatless Tuesdays” since Monday was the day of the week when he thought people were most likely to change their behavior (26). He ensured that Meatless Monday, with its memorable alliteration, was free and open-source in order to facilitate its dissemination. Lerner envisioned Meatless Monday being much like a national holiday – no one owned it.

**Figure 2:**
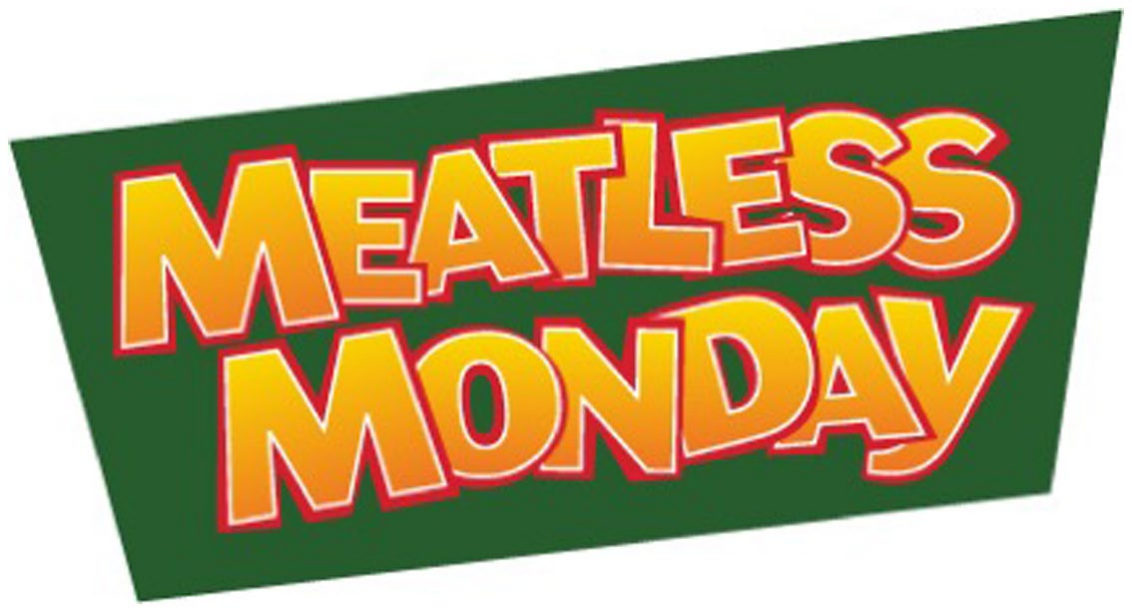
Meatless Monday campaign logo.

A Meatless Monday website (17) (Figure 2) was initiated to raise awareness about dietary recommendations (77) and to feature weekly meatless recipes to provide suggestions for alternatives to meat. Meatless Monday reached people with weekly periodicity and on a day when they were starting the week and most open to making healthy choices (78). Meat, as defined by the Meatless Monday campaign, included all livestock meat, i.e., chicken, pork, lamb, and beef. A year prior to the formal launch of the Meatless Monday campaign, Alfred Sommer, Dean of the Johns Hopkins Bloomberg School of Public Health [dean from 1990 to 2005], and Allan Rosenfield, Dean of the Columbia University Mailman School of Public Health, [dean from 1986 to 2008], elicited agreement from 28 deans of schools of public health to support Meatless Monday (79). Students in schools of public health across the US launched pilot campaigns on their campuses and in local communities from 2003 to 2006 (Pamela Berg, personal communication). Signs and posters and networking at national and international meetings extended the influence of Meatless Monday from 2003 to 2006 (Peggy Neu personal communication). As social media and internet websites grew from around 2005, the influence of Meatless Monday grew (Peggy Neu, personal communication).

In 2006, an online commercial food distributor, Fresh Direct, included Meatless Monday in its digital content (80). Boca Burger was the first food company to use Meatless Monday to promote their vegetarian products (81). Jenny Craig, Inc. added Meatless Monday to their weekly newsletter in 2007 (82). In 2008, Meatless Monday campaign staff attended the American College Health Association Meeting to promote the idea of Meatless Monday on college campuses, and several more campaigns were launched that year (Pamela Berg, personal communication).

In 2009, the Baltimore City Public Schools became the first school system in the country to observe Meatless Monday, a decision that was widely attacked by industry-aligned groups. The American Meat Institute countered with the falsehood that 75% of children were not getting enough protein (83). Michael Pollan, the food writer and health advocate, noted: “If Baltimore can pull this off, it will be a sign that the effort is worth making!” (84). Meatless Monday was subsequently taken up by entire school districts of other cities such as Boston, Buffalo, Detroit, Houston, Kansas City, Oakland, Philadelphia, Sarasota, and San Diego (85–87).

On Earth Day, April 22, 2009, Pollan endorsed Meatless Monday on the Oprah Winfrey Show and urged viewers to do the same. Oprah Winfrey subsequently cheered Meatless Monday on her show in February 1, 2011 and instituted Meatless Monday in the cafeteria of her Harpo Studios in Chicago (88). The food service company Sodexo, which provides meals for about 10 million people per day in the US, announced that they would offer Meatless Monday options on their corporate, healthcare, and college menus nationwide (89). The city of Aspen, Colorado, became the first city in the US to adopt Meatless Monday (90) and the following year, the Los Angeles City Council unanimously passed a resolution for making every Monday a Meatless Monday (91).

The Meatless Monday campaign began a full social media effort in 2009, disseminating teaching materials on how to implement Meatless Monday in cafeterias and providing meatless recipes on their website and through their newsletters (Peggy Neu, personal communication). Staff and researchers at the Center for a Livable Future were active in contributing materials for newsletters and blog posts (Peggy Neu, personal communication). The rise of food media, defined as discussions of food on social media and in newspapers and magazines, was a major driver of awareness for Meatless Monday (Peggy Neu, personal communication). Popular magazines and websites such as *Redbook*, *Women’s Day*, and *Huffington Post Green* provided recipes for Meatless Monday (Peggy Neu, personal communication). The *Washington Post* began a weekly blog on Meatless Monday (Kim O’Donnel, personal communication).

A controversy about Meatless Monday erupted at the USDA in July 2012. An interoffice newsletter was circulated within the USDA that mentioned green (environmental) initiatives at the Washington DC headquarters. It was suggested that the department’s cafeteria could participate in Meatless Monday, since “the production of meat, especially beef … has a large environmental impact … greenhouse gases and climate change are byproducts, as well as wasted energy resources.” (92). In addition, high meat consumption was mentioned as not being good for personal health (93). The beef industry and politicians from beef-producing states raised an outcry, which prompted the USDA to declare publicly that they would not observe Meatless Monday in their cafeteria (92). The debate over Meatless Monday at the USDA was brought further to national attention by the late-night comedian Stephen Colbert on August 2, 2012, season 8. Although Meatless Monday was not established in the Washington office cafeteria of the USDA, the controversy raised greater awareness of Meatless Monday across the US.

Prominent celebrities and health advocates such as Yoko Ono, Kate Moss, Richard Branson, Robin Roberts of the US television show, “Good Morning America,” and trainer Bob Harper of the US television show, “The Biggest Loser,” expressed support for Meatless Monday (94). In 2012, a video promoting Meatless Monday, produced by The Humane Society of the United States, was selected out of 11,000 entries around the world for a Telly Award, the premier award, established in 1979, that honors video and television (95). Other prominent celebrities to express support for not consuming meat on Monday included Beyoncé, Chris Martin, Reese Witherspoon, Tom Hanks, Rita Wilson, Orlando Bloom, Billie Eilish, and Ringo Starr (96). Meatless Monday received increasing attention from celebrity chefs, such as Katie Lee, Mario Batali, Wolfgang Puck, and John Fraser (97, 98). Over a 13-year period from 2005 to 2021, awareness of Meatless Monday increased from ~9% in 2005 to 38% by 2021 in a survey of a representative sample of US adults (99). The highest awareness rate was recorded in 2011, when 50% in the survey responded positively to the question, “Have you heard of Meatless Monday?” (100). Of those who were aware of Meatless Monday, 27% said that the campaign had influenced their decision to cut back meat (100). The New York City Public Schools began Meatless Mondays with the 2019–2020 school year (101). The Meatless Monday campaign, despite operating with an extremely limited advertising budget, spread on the merits of the idea and its uptake by the media and public.

## 7 Growing health concerns about high consumption of red and processed meat

When the Meatless Monday campaign began, total *per capita* meat consumption (beef, pork, poultry) in the US had increased by more than 60% from about 160 pounds per person in 1950 to more than 260 pounds per person by 2003 (102). Prior to 2003, public health experts were mostly focused on the relationship of saturated fats rather than meat consumption with cardiovascular disease (103). During the two decades that followed the founding of the Meatless Monday movement in 2003, new scientific reports linked high red and processed meat consumption with cardiovascular disease, diabetes, and cancer. High consumption of red and processed meat consumption was associated with an increased risk of stroke, coronary heart disease, and diabetes (105, 106). In 2016, the American Heart Association recommended to “limit intake” of processed meat (107). The dietary guidelines issued by the USDA continued to evolve from advice in 2000 to “Choose a diet that is low in saturated fat and cholesterol and moderate in total fat” (108) to include mention of the DASH Eating Plan in 2005 and 2010 in which a healthy diet pattern “… feature[s] less red and processed meat and more seafood than typical American diets” (109). In 2020, the *Dietary Guidelines for Americans* stated that dietary patterns with positive health outcomes were characterized by “lower consumption of red and processed meats,” and that “About three-quarters of Americans meet or exceed the recommendations for meats, poultry, and eggs.” (110).

In addition, higher dietary intake of red meat and processed meat was associated with greater risk of colorectal cancer (111, 112). In October 2015, the expert working group of the International Agency for Research on Cancer, the cancer agency of the World Health Organization, reviewed the scientific evidence and determined that the consumption of processed meat was “carcinogenic to humans” and that the consumption of red meat was “probably carcinogenic to humans” (112–115). The American Cancer Society issued guidelines in 2020 for a healthy dietary pattern that “limits or does not include red and processed meat” (116).

The link between red and processed meat with some types of cancer prompted some healthcare writers to recommend Meatless Monday to reduce the risk of cancer (117, 118). On NBC News, Allison Van Dusen, senior editor at the Mayo Clinic, touted the potential health benefits in heart disease and cancer risk of cutting back on meat through participation in Meatless Monday (119). The city of Vancouver observed Meatless Monday on June 10, 2013, partly to raise awareness about how scientific evidence showed that a diet high in processed and red meat increases the risk of colon cancer (104). The large US healthcare provider, Patient First, advocated Meatless Monday to reduce the chance of cancer and cardiovascular disease (120).

## 8 Meat production identified as major adverse cause of climate change

In 2006, the Food and Agricultural Organization (FAO) of the United Nations issued a seminal report regarding the impact of livestock production on the environment (121). Their analysis showed that the livestock sector accounted a substantial amount of greenhouse gas emissions (GHG) and global water use and was a major factor in deforestation and loss of species (121). The FAO report concluded: “… The impact of livestock on the local and global environment is so significant that it needs to be addressed with urgency. Information, communication and education will play critical roles in the promotion of an enhanced willingness to act. Consumers, because of their strong and growing influence in determining the characteristics of products, will likely be the main source of commercial and political pressure to push the livestock sector into more sustainable forms” (121). Further studies showed that the water footprint of beef production greatly exceeds the production of other major plant and animal foods (122).

An updated analysis by FAO showed that livestock, which include primarily ruminant (cattle, lamb) and monogastric (pigs, chicken) animals, account for 14.5% of total human-induced GHG emissions, with cattle contributing 65% of total livestock GHG emissions (123). In 2015, at the United Nations Climate Change Conference (Conference of the Parties [COP] 21), an international treaty known as the Paris Agreement was ratified to hold “the increase in the global average temperature to well below 2°C above pre-industrial levels” and pursue efforts “to limit the temperature increase to 1.5°C above pre-industrial levels.” If no changes are made to the current trends in global food systems, even with total reduction of fossil food emissions, it will not be possible to achieve the 1.5°C limit of the Paris Agreement (124). Scientists made suggestions how changes in food consumption could potentially lower GHG emissions. Simple dietary substitutions of chicken for beef would reduce GHG emissions by an estimated 48% and water use by 30% in US consumers (125). Substituting beef with beans in US diets could reduce GHG production even further and free up >40% of US cropland (126). In 2019, the EAT-Lancet Commission proposed a global reference diet, the planetary health diet, that was based on optimal nutrition for health and ecological sustainability (127). The planetary health diet emphasizes whole grains, plant proteins, fruits and vegetables, and modest amounts of meat and dairy (127). An analysis of country-specific dietary shifts in 140 countries by Kim and colleagues showed that adoption of a meatless day worldwide would reduce GHG and water footprints in high income countries but overall would be associated with a small global net increase in GHG and water footprints due to shifts from nutritionally inadequate diets in lower and middle-income countries (128). In the modeling of a meatless day diet, meat (defined as beef, pork, lamb, and goat) was included in the six of seven days of the diet. The consumption of meat is low in countries such as India, Indonesia, and Pakistan, thus, in this model, a meatless day diet projected an increase in diet-related GHG production in countries with low consumption of meat (129).

The *Washington Post* blogger and cookbook author, Kim O’Donnel, got actively involved with Meatless Monday after she heard a speech delivered by UN climate expert and chairman of the Intergovernmental Panel on Climate Change (IPCC), Rajendra Pachauri, in which he said that the most important thing one could do for the planet is to reduce meat consumption (129). Paul McCartney decided to become involved with Meatless Monday after he read the 2006 report by FAO (121) regarding the large contribution of livestock production to greenhouse gas emissions (130). The 2014 IPCC report was a stimulus for reducing meat in the diet and inspired some to start with Meatless Monday (131).

## 9 Connecting public health, the environment, and animal welfare

The One Health movement, a collaborative effort of multiple disciplines to attain optimal health for people, animals and our environment (132), grew in prominence from 2003 to 2023. One Health emphasizes the interrelationships between human, animal, and environmental health (133). The American Veterinary Medical Association formed a task force on the One Health concept that resulted in a report, *One Health: A New Professional Imperative*, in 2008 (134). In 2009, a One Health office was established by the Centers for Disease Control and Prevention. Animal science specialists and veterinarians have emphasized that One Health is a platform for improving the welfare and health of animals raised in industrialized agriculture (132, 135).

In 2008, the Pew Commission on Industrial Farm Animal Production issued a report that assessed the industry’s impact on the public’s health, the environment, rural communities, and animal health and well-being. The report shed light on the industry’s intensive practices in food animal production (meat, eggs and dairy), including the overuse of antibiotics, generation of highly concentrated hazardous wastes, confinement of farm animals, and the adverse environmental and health impacts on communities (136). To accommodate the industrial model, food producing animals and their environments are engineered to ensure high productivity. The animal welfare aspects of this report built upon earlier work, such as Ruth Harrison’s *Animal Machines* (1964) (137), Peter Singer’s *Animal Liberation* (1975) (138), and the Brambell report which codified what became known as the Five Freedoms, a minimal set of moral rights for farm animals to protect them against unnecessary suffering (139).

In 2016, the United Nations Committee on World Food Security addressed the environmental, economic, social and equity dimensions of the livestock component of agricultural systems. Recognizing the connections among farm animal welfare and other dimensions of agricultural development, the report recommended that action be taken to improve animal welfare and intensive livestock systems: “The biggest welfare wins can be achieved on farm, where animals spend most of their time. For example, moving from close confinement systems such as sow stalls (gestation crates) to group housing systems, and cage-free rather than battery cage layer hen production. Alongside legislative requirements in the EU, many large food companies now require commitments to phase in improved animal welfare in their supply chains, including major food service multinationals and producers” (140).

The Humane Society of the United States started a Meatless Monday initiative in 2011, when they hired Kristie Middleton. By 2016, there were 15 people working on the team, and they helped get 200 school districts to participate in Meatless Monday (141). When the University of Notre Dame adapted Meatless Monday in 2012, Kenny Torrella, coordinator at the Humane Society of the United States, remarked, “If each American chose meatless options just 1 day a week, more than a billion animals would be spared from factory farms each year …” (142).

## 10 Meatless Monday becomes a global movement

**Figure 3:**
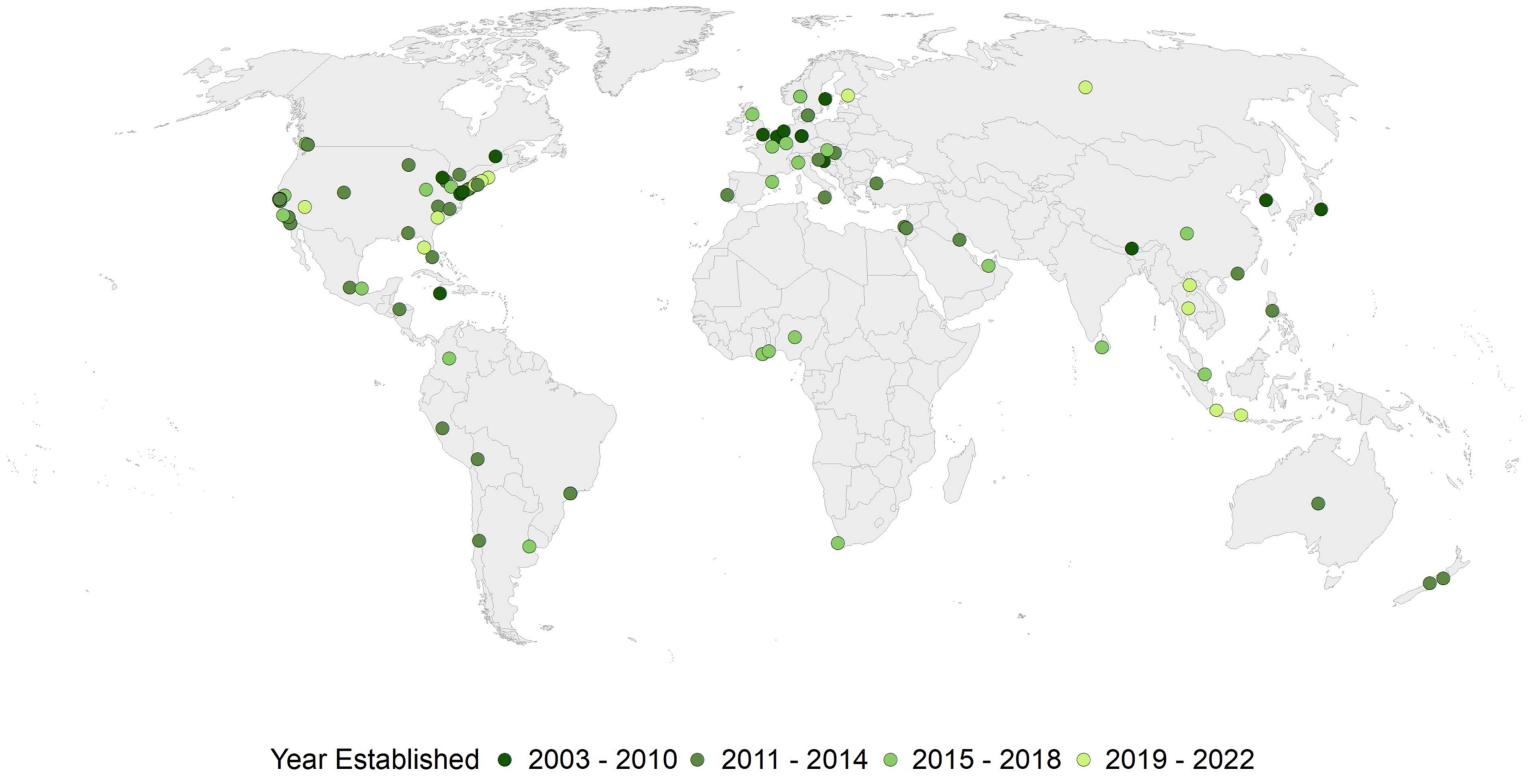
Global map of Meatless Monday activities, 2003–2022. Location of Meatless Monday activities in locations worldwide by time intervals in years specified.

As global awareness of the environmental and health issues surrounding high meat production and consumption has grown since 2003, the value and relevance of Meatless Monday has increased as well. Meat-Free Monday campaign was initiated in the United Kingdom by Sir Paul McCartney and his daughters Mary and Stella in 2009 (143). The same year, Belgium launched Donderdag Veggiedag (Thursday Veggie Day) (144). With financial support of the Flemish government, vegetarian restaurants blossomed in the city of Ghent, and it soon became the reputed “veggie capital of Europe” (145). The Meatless Monday campaign, as “Segunda Sem Carne,” was initiated in Brazil by the Sociedade Vegetariana Brasileira in 2009 (146). Meatless Mondays Australia was founded in 2009 by Deb Robbins and Vinita Chopra. They noted, “Meatless Monday Australia represents a creative, practical avenue for people around the world to help save the planet and its inhabitants. Not everyone can buy an eco-friendly car, some people may not have a garden, it may not be practical or safe for others to travel by public transport or on foot, but eating vegetarian meals 1 day a week can make a world of difference.” (147). Meatless Mondays spread to over 40 countries (Figure 3), including the Philippines (148), Malaysia (149), United Arab Emirates (150), Singapore (151), and Israel (152).

The Norwegian Army adopted Meatless Mondays in 2013 (153). In order to raise consciousness about excessive meat consumption and soaring obesity rates in Argentina, the Casa Rosada, the presidential palace, instituted Meatless Mondays in 2017 (154). The Meatless Monday campaign conveyed the message “Less Meat = Less Heat” at the United Nations’ Climate Change Conference (COP21) in Paris in December 2015 (155). Several Canadian universities, including McGill, Dalhousie, Queen’s, and Langara College, adopted Meatless Monday (156). Colleges, high schools, and hospitals in Vancouver joined the Meatless Monday initiative in 2016 (157). In 2019, actors and celebrities, including Juliette Binoche, called for the adoption of “Lundi Vert” (Green Monday) as a meatless day in France (158, 159). The network of university restaurants across France started participating in 2019 by offering wider vegetarian options to fit the objectives of the “Lundi Vert” campaign (160). The Green Schools program, run by An Taisce, Ireland’s leading national operator for environmental education programs, advocated Meatless Monday for secondary schools in Ireland (161).

## 11 Impact of the Meatless Monday campaign

Despite relatively high name recognition, there have been only a limited number of studies that have assessed participation in Meatless Monday or the impact of the Meatless Monday upon awareness of the health and environmental impact of meat consumption, attitudes toward meat consumption, or change in dietary habits. In France, a large cross-sectional survey showed that those who participated in “Green Monday” were more likely to be women, more educated, and with higher self-rated affluence compared with controls (162). A community survey of Meatless Monday participation in Bedford, New York showed that participants were more likely to be women and with higher income but no significant difference in education level compared with controls (163). Meatless Monday was evaluated for perceived message effectiveness in an online randomized study of 1,244 US adults aged 18 years and older (164). Subjects were randomized to control messages, Meatless Monday health-focused messages, or Meatless Monday environmentally-focused messages. Those exposed to the Meatless Monday messages showed greater intention to reduce meat consumption compared with the control group (164).

## 12 Future research

Future research could help address major gaps in knowledge regarding the impact of Meatless Monday on health, the environment, policymakers, and the food industry. The framing of the message about meat reduction can vary widely (165). What messaging by Meatless Monday has been the most influential? Behavior change is influenced by the need of people for information (166). Can the messaging of Meatless Monday be tested and improved to reduce consumption more effectively? Can response inhibition training (167) be applied to Meatless Monday to help people reduce their meat intake? The long-term effects of behavior change with Meatless Monday participants are not well characterized. For example, what proportion of participants in Meatless Monday eventually end up as flexitarians or vegetarians? What is the environmental impact when large school systems, such as New York City public schools as an example, observe Meatless Monday? What is the impact of large-scale implementation of Meatless Monday in public schools on food providers? What are the primary motivating factors for people to participate in Meatless Monday, and how does this differ around the world? Case studies could shed insight on underlying factors for either the success or failure of Meatless Monday initiatives and food policy around the world. If future research shows that participation in Meatless Monday leads to significant reductions in meat consumption, such dietary shifts have implications for health, the environment, and the food industry.

## 13 Discussion and conclusion

The concept of Meatless Monday was based upon meatless days in World War I and II when a large proportion of the US population widely practiced meatless days to conserve meat for the Allies and the troops. Based upon our historical review, we summarize the similarities and differences between the meatless days in World War I and II with the Meatless Monday campaign in terms of motivating factors, means of communication, scientific findings about meat, and other factors in Table 1. Antagonism to meatless days was relatively muted during World War I and II. The US meat industry, which has been a vociferous opponent of the current Meatless Monday campaign, uses an industrial animal model that did not exist during the first half of the twentieth century. With the Meatless Monday campaign, the motivations for observing a meatless day shifted to varied concerns about health, animal welfare, and the environment as seen, for example, in work by *Washington Post* food blogger Kim O’Donnel, involvement by Paul McCartney and the founding of Meat-Free Monday, and the involvement of the Humane Society of the United States in getting US public schools involved in Meatless Monday.

**Table 1:** Approaches to meatless days in World War I and II and now.

	World War I and II	Meatless Monday campaign
World population	1.7 billion (1914) 2.0 billion (1939)	6.4 billion (2003) 8.0 billion (2023)
Meat definition	Beef, lamb, pork	Beef, lamb, pork, chicken
Motivating factors	Provide food for Allies Provide food for the troops Patriotism	Personal health Planetary health Animal welfare
Communication	Newspapers and magazines Signs and posters radio Door-to-door pledge campaigns	Newspapers and magazines Signs and posters Television Websites Social media
Local leadership	Mayors Restaurant associations	Mayors School administrations Food distributors
People of influence	Politicians Movie stars	Politicians Movie stars Rock stars Food writers Celebrity cooks Television talk show hosts
Scientific findings on meat	A source of protein that could be replaced by legumes	Consumption linked with heart disease, diabetes, cancer
Advice for households	Cookbooks Recipes in magazines	Cookbooks Recipes in magazines Recipes in social media
Antagonists	Not very vocal	Meat industry Politicians from meat-producing states

The strengths of this review are the involvement and contributions of three individuals who played a role in the early years of the Meatless Monday campaign (Peggy Neu, Pamela Berg, Shawn MacKenzie) and a comprehensive search of newspaper and periodical databases. A limitation of this review is that some parts of the world were not as comprehensively reported due to a focus on English, Spanish, Portuguese, and French and the paucity of newspaper and periodical databases in other languages and from low-and-middle-income countries and other parts of the world.

Meatless Monday grew steadily from 2003 to 2023 since its inception through advocacy by food writers, talk show hosts, and celebrity chefs, and through participation by schools, cities, restaurants, and institutions worldwide. During the same period, there were growing concerns about the environmental impact of meat production and adverse health consequences of high meat consumption. From numerous professional and scientific communities, several important findings and positions also contributed greater understanding of the impacts of high meat consumption and the practices associated with the industrial model. FAO linked livestock production with high GHG emissions. Hundreds of scientific papers showed that meat consumption increased the risk of cardiovascular disease, diabetes, and cancer. The World Health Organization issued a warning that processed meat was carcinogenic. US dietary guidelines emphasized a healthy dietary pattern that was high in fruit and vegetable and limited in meat consumption. International organizations expressed increasing concerns for farm animal welfare. Meatless Monday grew from relatively humble origins to a highly recognized worldwide movement that continues to raise awareness of healthy alternatives to meat consumption for personal and planetary health.

## Author contributions

RS: Conceptualization, Investigation, Methodology, Writing – original draft, Writing – review & editing. PN: Investigation, Writing – review & editing. PB: Investigation, Writing – review & editing. JH: Methodology, Visualization, Writing – review & editing. SM: Funding acquisition, Writing – review & editing. RR: Conceptualization, Data curation, Project administration, Writing – review & editing.
